# Non-Invasive Sensor-Based Estimation of Anterior-Posterior Upper Esophageal Sphincter Opening Maximal Distension

**DOI:** 10.1109/JTEHM.2023.3246919

**Published:** 2023-02-20

**Authors:** Yassin Khalifa, Amanda S. Mahoney, Erin Lucatorto, James L. Coyle, Ervin Sejdić

**Affiliations:** Department of Biomedical EngineeringCairo University 63526 Giza 12613 Egypt; Department of Electrical and Computer EngineeringSwanson School of EngineeringUniversity of Pittsburgh 6614 Pittsburgh PA 15260 USA; Case Western Reserve University School of Medicine 12304 Cleveland OH 44106 USA; University Hospitals Harrington Heart and Vascular Institute Cleveland OH 44106 USA; Department of Communication Science and DisordersUniversity of Pittsburgh 6614 Pittsburgh PA 15260 USA; Department of OtolaryngologyUniversity of Pittsburgh 6614 Pittsburgh PA 15260 USA; The Edward S. Rogers Sr. Department of Electrical and Computer EngineeringFaculty of Applied Science and EngineeringUniversity of Toronto 7938 Toronto ON M5S 1A1 Canada; North York General Hospital 8613 Toronto ON M2K 1E1 Canada

**Keywords:** Swallowing, accelerometry, vibrations, cervical auscultation, dysphagia, aspiration, upper esophageal sphincter, attention mechanisms, signal analysis, deep learning, supervised learning, recurrent neural networks, GRU

## Abstract

Objective: Dysphagia management relies on the evaluation of the temporospatial kinematic events of swallowing performed in videofluoroscopy (VF) by trained clinicians. The upper esophageal sphincter (UES) opening distension represents one of the important kinematic events that contribute to healthy swallowing. Insufficient distension of UES opening can lead to an accumulation of pharyngeal residue and subsequent aspiration which in turn can lead to adverse outcomes such as pneumonia. VF is usually used for the temporal and spatial evaluation of the UES opening; however, VF is not available in all clinical settings and may be inappropriate or undesirable for some patients. High resolution cervical auscultation (HRCA) is a noninvasive technology that uses neck-attached sensors and machine learning to characterize swallowing physiology by analyzing the swallow-induced vibrations/sounds in the anterior neck region. We investigated the ability of HRCA to noninvasively estimate the maximal distension of anterior-posterior (A-P) UES opening as accurately as the measurements performed by human judges from VF images. Methods and procedures: Trained judges performed the kinematic measurement of UES opening duration and A-P UES opening maximal distension on 434 swallows collected from 133 patients. We used a hybrid convolutional recurrent neural network supported by attention mechanisms which takes HRCA raw signals as input and estimates the value of the A-P UES opening maximal distension as output. Results: The proposed network estimated the A-P UES opening maximal distension with an absolute percentage error of 30% or less for more than 64.14% of the swallows in the dataset. Conclusion: This study provides substantial evidence for the feasibility of using HRCA to estimate one of the key spatial kinematic measurements used for dysphagia characterization and management. Clinical and Translational Impact Statement: The findings in this study have a direct impact on dysphagia diagnosis and management through providing a non-invasive and cheap way to estimate one of the most important swallowing kinematics, the UES opening distension, that contributes to safe swallowing. This study, along with other studies that utilize HRCA for swallowing kinematic analysis, paves the way for developing a widely available and easy-to-use tool for dysphagia diagnosis and management.

## I. Introduction

Dysphagia, or swallowing dysfunction, occurs secondary to a variety of illnesses, disorders and traumatic injuries that disrupt the well coordinated mechanism of swallowing. Dysphagia is a primary cause of aspiration pneumonia which is associated with higher mortality rates than non-aspiration pneumonia [1], [2]. Swallowing impairments that lead to dysphagia are usually identified by the temporospatial kinematic analysis of videofluoroscopy (VF) images to determine the severity of the underlying condition and the best course of intervention [3], [4]. Temporospatial kinematic analyses of VF studies performed within clinical and research settings, include measurements of swallow biomechanical events that directly contribute to the safe execution of swallowing, including the upper esophageal sphincter (UES) opening [5], [6], [7].

The UES is a muscular valve which permits the transfer of food and/or liquid (i.e., the bolus) from the pharynx to the esophagus during swallowing. The UES opening process involves multiple stages including relaxation, opening, distension, collapse and closure, and relies on precise timing to guarantee complete passage of the bolus into the esophagus without the accumulation of pharyngeal residue. UES opening is facilitated by traction forces produced by the combination of suprahyoid muscular contraction and anterior-superior hyolaryngeal excursion [5], [7]. These traction forces, bolus propulsion and the traction forces applied to the anterior wall of UES by relaxation of the pharyngeal elevator muscles contribute to UES distension [3]. Delayed UES opening and/or reduced UES distension may result in pharyngeal residue and increased risk of airway invasion, via laryngeal penetration and/or aspiration into the trachea and lungs [3], [8], [9], [10], [11]; however, there is limited evidence in the literature regarding the direct/independent association between UES dysfunction and aspiration [3], [12].

Clinical assessment of UES function is performed via multiple modalities including the videofluoroscopy swallowing study (VFSS), fast pharyngeal CT/MRI, fiberoptic endoscopic evaluation of swallowing (FEES), and non-imaging instrumental tools such as electromyography (EMG) and high resolution pharyngeal manometry (HRM) [13], [14]. VFSS and HRM are the most frequently used modalities for the assessment of UES function during swallowing [3]. Previous studies showed multiple limitations and challenges for using the previously listed modalities to evaluate the UES function such as radiation exposure and low resolution of VFSS, invasiveness in HRM and FEES, and the need for clinical expertise for both conducting and interpreting the exams. Moreover, these exams are vulnerable to subjectivity in judgment and human error and are not available in all clinics which can delay the diagnosis of many patients, putting them at risk for complications related to dysphagia [13].

There is high demand for a low cost, noninvasive and objective tool to provide an equivalent diagnostic value for dysphagia as the image-based swallow assessment modalities. Such a tool could provide real-time insights about the biomechanical properties of the swallow to help guide the diagnosis and rehabilitation of dysphagia. High resolution cervical auscultation (HRCA) is a sensor-based technology recently proven helpful to perform real-time temporospatial kinematic measurements of swallowing as accurately as measurements done by expert human judges in VFSS [13], [15]. HRCA combines signal processing, machine learning and time series analysis techniques to temporally localize swallow kinematic events such as laryngeal vestibule closure and reopening, and UES opening and closure [13], [15], [16], [17], [18]. HRCA has not only been effective in the temporal localization of swallow kinematic events, but also in performing spatial swallow measurements such as tracking hyoid bone displacement with high accuracy as compared to measurements by expert judges on VFSS [19], [20]. Further, strong associations exist between HRCA signals and other swallow spatial measurements such as the anterior-posterior (A-P) UES opening maximal distension [21]. Using HRCA to quantitatively measure the A-P UES opening maximal distension has not yet been addressed or implemented.

As previously mentioned, HRCA was used to temporally identify UES opening timing by implementing a hybrid convolutional recurrent neural network (CRNN), which takes the raw HRCA signals as input [13]. This CRNN employed convolutional networks (CNNs) in the first layers for local feature extraction from the raw signals and reduction of the number of time steps through which the error signals propagate in the network. The CNN was followed by a recurrent neural network (RNN), which has the ability to model temporal dependencies along the localized features extracted by the CNN [13], [22]. This network achieved high accuracy in detection of UES opening time when compared to manual measurements performed by expert judges in VFSS. The UES opening detection study and previous studies that associated HRCA signals with the A-P UES opening maximal distension have guided the endeavor of this study to build a deep learning platform that uses HRCA signals, hybrid CRNNs and attention mechanisms to accurately measure the A-P UES opening maximal distension during swallowing.

We investigated the possibility of using HRCA signals to non-invasively estimate the A-P UES opening maximal distension during swallowing. The multi-channel HRCA signals were fed into a hybrid CRNN that employs attention to focus only on the signals during which the UES was open. This algorithm, along with the UES opening detection algorithm, offers a complete picture of the efficiency and duration of the UES opening during swallowing, which can be used by clinicians to determine factors contributing to possible adverse swallowing conditions such as the possibility of residue formation and/or penetration and aspiration.

## II. Methods

### A. Study Design and Clinical Protocol

This study was approved by the institutional review board of the University of Pittsburgh. All participating subjects provided informed written consent prior to enrollment, including consent to publish. We collected data from 133 patients (93 males, 40 females, age: 64.3 ± 13.2) with a variety of diagnoses, with suspected dysphagia. Thirty-seven subjects were diagnosed with stroke while the other 96 patients were admitted due to other medical conditions unrelated to stroke such as neurodegenerative diseases and lung transplant. The patients underwent an oropharyngeal swallowing function evaluation using VF at the University of Pittsburgh Medical Center Presbyterian Hospital (Pittsburgh, PA, USA).

This study was conducted as a part of a standard clinical procedure rather than a controlled research protocol. As a result, the swallowing assessment was modified according to the patient’s status and condition, which may have altered the volume and consistency of the boluses, the mood of administration (e.g., cup or spoon), and the patient’s head position during swallowing. The administered boluses included the following consistencies: thin liquid (Varibar thin, Bracco Diagnostics, Inc., < 5 cPs viscosity), mildly thick liquid (Varibar nectar, 300 cPs viscosity), puree (Varibar pudding, 5000 cPs viscosity), and Keebler Sandies Mini Simply Shortbread Cookies (Kellogg Sales Company). The boluses were administered by the speech language pathologist conducting the exam or were self-administered by the patient. Four hundred and thirty-four swallows (203 from stroke-diagnosed patients and 230 from patients with other non-stroke conditions) were collected and analyzed in this study.

### B. Data Acquisition

The experimental setup of this study is like that of our previous research on UES opening [13]. Subjects were comfortably seated and VFSS was conducted in the lateral plane using a Precision 500D system (GE Healthcare, LLC, Waukesha, WI) at a pulse rate of 30 pulses per second [23]. The VFSS feed from the x-ray machine was connected to the data acquisition workstation through an AccuStream Express HD video card (Foresight Imaging, Chelmsford, MA) that digitized the video feed at a resolution of $720\times 1080$ and a sampling rate of 60 frames per second (FPS). Swallowing vibrations were collected simultaneously with VFSS through a tri-axial accelerometer (ADXL 327, Analog Devices, Norwood, Massachusetts) that was attached to the skin overlying the cricoid cartilage using an adhesive tape [15]. The accelerometer’s axes were aligned to gather vibrations in the anterior-posterior (A-P), superior-inferior (S-I), and medial-lateral (M-L) directions. The signals were fed into the same acquisition workstation as the VFSS feed through a 6120 DAQ (National Instruments, Austin, Texas) and digitized in a rate of 20 kHz. The collection of streams from the VFSS and the accelerometer was synchronized using LabView (National Instruments, Austin, Texas). The accelerometer signals were later downsampled to 4 kHz to smooth out transient noise and measurement errors [13].

### C. VFSS Image Analysis and UES Distension Expert Measurement

VFSS videos were segmented into individual swallow segments by tracking the bolus to determine the onset and offset of pharyngeal swallowing. The onset of the swallow was defined as the frame in which the bolus head passed the ramus of the mandible, and the offset of the swallow was defined as the frame in which the hyoid bone returned to its lowest resting position after clearance of the bolus tail through the UES [15], [24], [25]. The time of UES opening and closure were determined for each swallow in the segmented videos. All judges who performed swallow segmentation and UES opening and closure ratings were trained to perform swallow kinematic measurements in VFSS and established a priori intra- and interrater reliability with ICC’s over 0.99. Judges also maintained similar reliability ICC’s throughout measurements on 10% of the swallows. Raters were blinded to all swallow information and the subject’s diagnosis to avoid bias.

**Fig. 1.**
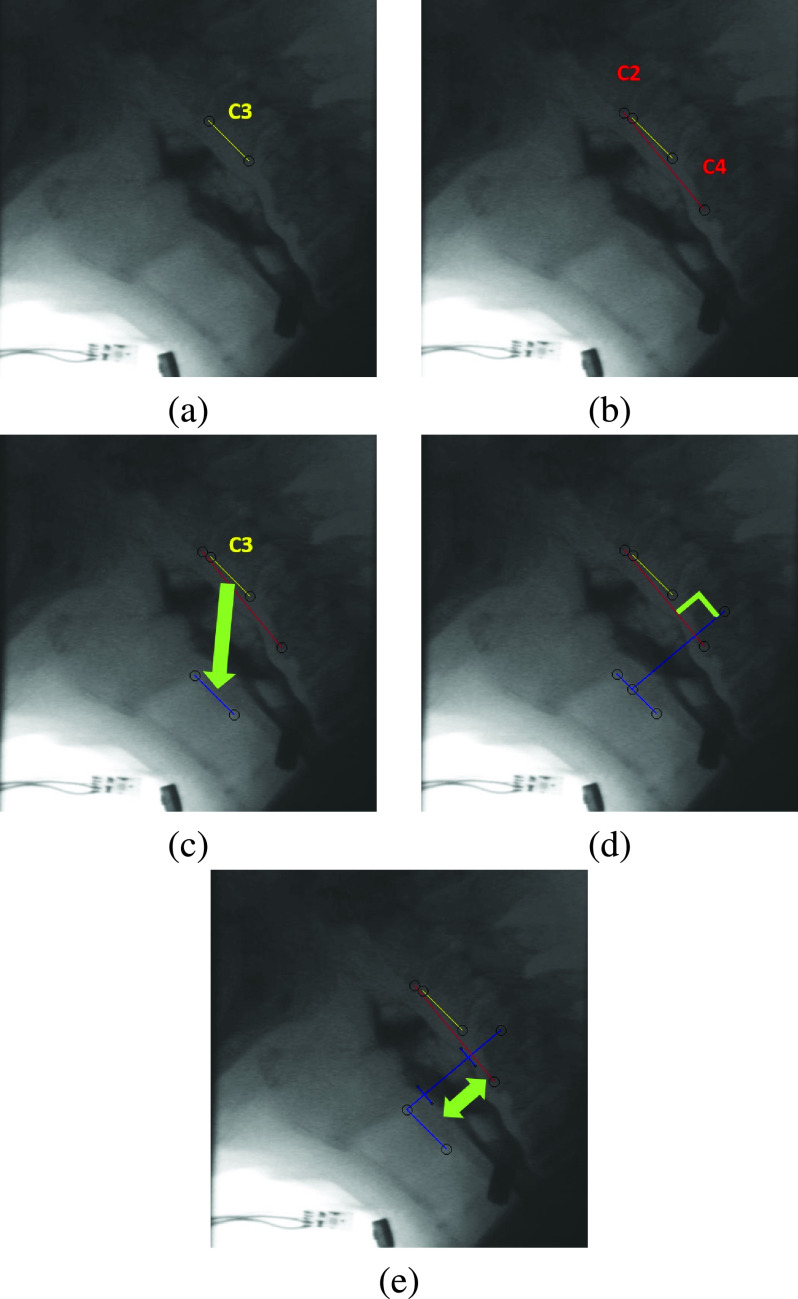
Graphical representation of measuring the A-P UES opening maximal distension using the aforementioned software: (a) C3 height is marked with a yellow line; (b) C2-C4 height is marked with a red line to be used as the pseudo vertical axis for measurements and as an anatomical scalar for the subject’s height; (c) The repositioned C3 segment with its top point anchored to the superior-posterior border of tracheal air column; (d) The pseudo horizontal axis of measurements is generated as the long blue line perpendicular to C2-C4 line. The anterior end of the pseudo horizontal axis slides between the end points of the anchored C3 segment; (e) The pseudo horizontal axis is vertically adjusted to the location of the UES maximal distension along C2-C4, and the anterior and posterior walls of the UES are marked with two short blue lines perpendicular to the pseudo horizontal axis. The A-P UES opening maximal distension is measured as the distance between the two short blue lines.

To measure the A-P UES opening maximal distension, judges selected the frame of maximal anterior-superior hyoid bone displacement in the pharyngeal phase of swallowing. The UES maximal distension usually happens at, shortly before or shortly after the frame of the maximal hyoid bone displacement, so judges measured the UES distension at the frame of the maximal hyoid bone displacement, 2-3 frames before and 2-3 frames after (5-7 frames in total). The A-P maximal distension was calculated using all measured frames [7], [21], [26]. Judges measured selected frames using a protocol and a software developed in our lab [21]. The protocol was as follows:

1) The height of the third vertebral unit (C3) was used to standardize the location of the superior and inferior limits of the UES. The UES, defined as the region of the proximal esophagus, was quantified in previous studies as coursing 1.3 cm inferiorly from the base of the true vocal folds [26]. The height of the third vertebral unit ranges from 1.11-1.37 cm in adults based on midsagittal x-ray measurements [27]. Therefore, the height of the C3 was marked by a yellow line that extended from the anterior-superior corner to the anterior-inferior corner of the C3 Fig. 1 (a).
2) The length of the C2-C4 segment was used as a pseudo vertical axis to compensate for head and neck rotation. The length of the C2-C4 segment was marked by a red line that extended from the anterior-inferior corner of the second vertebral unit (C2) and the anterior-inferior corner of the fourth vertebral unit (C4) (Fig. 1 (b)) [28]. The length of the C2-C4 segment was also used as a representative scalar for the subject’s height [28].
3) The yellow line representing the C3 height from step 1 was repositioned and anchored to the notch formed by the superior border and posterior wall of the tracheal air column, as shown in Fig. 1 (c).
4) The software automatically generated a long blue line perpendicular to the C2-C4 segment. This line was used as the A-P axis for UES distension measurement rather than using an arbitrary horizontal axis that could result in inaccurate measurements caused by head and neck rotation. The blue line could be repositioned by judges between the superior and inferior ends of the newly placed C3 segment from step 3 to the location of maximal A-P distance of the UES opening (Fig. 1 (d)).
5) The judges marked the anterior and posterior points of the open UES on the blue A-P axis generated in step 4. Upon marking these two points, the software generated two short blue lines to indicate the anterior and posterior walls of the UES opening (Fig. 1 (e)).
6) The software returned the coordinates of the anterior and posterior wall points marked in step 5 as an output to be used for the calculation of the A-P UES opening maximal distension.

The measured A-P UES opening maximal distension value was divided by the length of the C2C4 segment to standardize and compensate for the height of each patient. The C2C4 segment length represents a part of the vertebral column which corresponds with the patient’s height, so we used this as a standardization procedure for the A-P UES opening maximal distension value as followed in multiple studies [29], [30], [31].

### D. Signal Preprocessing

The pharyngeal swallow event is usually temporally accompanied by various other physiological events such as breathing and coughing, which also contribute to the collected vibratory and acoustic signals by the used sensors [32]. As a first preprocessing step performed on the collected signals to reduce such confounding noise sources, the signals which were accrued originally at 20 kHz, were downsampled to 4kHz. The 4 kHz frequency was chosen based on multiple factors including the fact that maximum swallowing frequency components reported in the literature (max energy frequency below 100 Hz and central frequency below 300 Hz) and that the top frequency component passed by the accelerometer on-chip low-pass filter is with 1600 Hz [13], [33], [34], [35], [36]. The downsampling step was performed through anti-aliasing low pass filtration to limit the frequency response followed by reduction of number of samples to match the new sampling frequency.

Zero-input response of the of the microphone and accelerometer, known as device noise, were recorded and modeled via a 10 $^{\mathrm{ th}}$ order modified covariance auto-regressive model [34], [37]. The order of the model was estimated using the Bayesian information criterion [34]. Four finite impulse response (FIR) filters were constructed based on the coefficients of the auto-regressive models to eliminate the device noise from each of the sensors [34]. Afterwards, fourth-order least-square splines were utilized to remove motion artifacts and low-frequency noise [38], [39]. The splines used a number of knots equivalent to $\frac {N\times f_{l}}{f_{s}}$, where $N$ is the data length and $f_{s}$ is the sampling frequency. $f_{l}$ is known as the lower sampling frequency and it is proportional to the frequency associated with motion artifacts. The values of $f_{l}$ were estimated and optimized in previous studies [38]. Finally, wavelet denoising with tenth-order Meyer wavelets and soft thresholding were used to reduce the effect of other noise sources of higher frequencies [40]. Threshold was estimated using $\sigma \sqrt {2\log N}$, where $N$ is the number of samples and $\sigma $ is the estimated standard deviation of the noise (calculated through down-sampling the wavelet coefficients) [40], [41].

### E. Design of the Deep Prediction Model

**Fig. 2.**
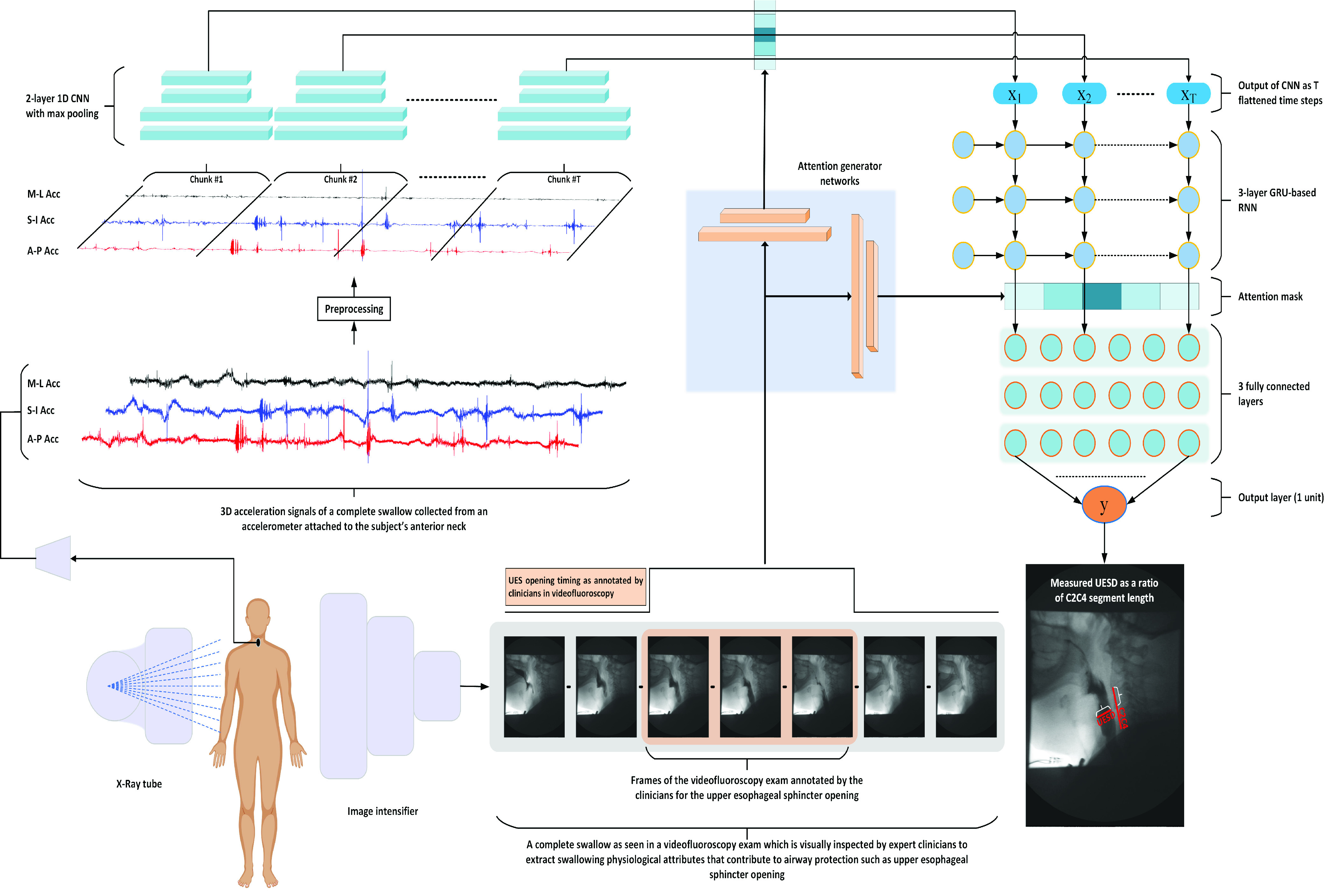
The architecture and data flow in the UES opening maximal distension prediction system. The lower left corner illustrates the first step in the experimental process in which HRCA signals and VFSS were collected simultaneously from the subject. Then, the 3-channel HRCA acceleration signals from each swallow were denoised and split into equal chunks of 66 samples (equivalent to 1 VF frame). The architecture of the 1D CNN was comprised of two layers, the first applied 16 filters on each channel and produced 48 channels. The attention generator networks are depicted in the center of the figure. The attention networks (two fully connected layers) took the UES opening mask as input, which generated the attention masks for the CNN and the RNN output. $x_{1:T}$ is the output train from the CNN for chunks ( $1:T$) after being masked by the generated attention and fed into the RNN units. Each unit in the RNN was built based on the gated recurrent unit design (GRU). The architecture of the 3-layer RNN used for time sequence modeling is shown in the upper right corner of the figure. The output sequence from the last layer of the RNN ( $\hat {y}_{1:T}$) was flattened and masked by the attention and fed into the first fully connected layer. (h) A diagram of the 3 fully connected layers (each of 128 units) used to combine the features coming out of the RNN is depicted in the right middle Section of the figure, under which is the output layer, composed of 1 unit ( $y$) that resembles the UES opening maximal A-P distension prediction as a ratio of the C2C4 segment length.

The design of the network implemented in this study, was fine-tuned based on an experimental approach and following the best practices that achieved high performance in similar problems [13], [42], [43]. Our network design was similar to one that detected UES opening duration in HRCA signals, which adopted a hybrid CRNN that works directly on the raw HRCA vibrational signals [13]. In this study, we changed the original network implemented in [13] based on the knowledge that HRCA signals are strongly correlated with the values of the A-P UES opening maximal distension rather than the duration of the swallow [21]. Therefore, we added an attention mechanism that was built and trained using a zeros/ones mask that resembles the UES opening duration labeled by expert judges as shown in the lower middle Section of Fig. 2.

The general network architecture was comprised of a 1D convolutional neural network, which included two convolutional layers with a max pooling layer in between. Both convolutional layers were followed by a rectified linear unit (ReLU). The first convolutional layer applied 16 “ $1 \times 5$” filters per channel. The max pooling layer consisted of a window of size 2 with 2 strides. The last convolutional layer was identical to the first layer except except for using only one filter per channel. The longest swallow segment in the collected data lasted around 1500 msec (90 frames of VFSS @60FPS), so the signals of each swallow were divided into smaller chunks 16.67 msec in length ( $\equiv ~1$ frame in VFSS or 66 samples in signals). Each chunk from the signals consisted of 3 channels of HRCA acceleration signals which made the dimensions 66 samples $\times3$ channels.

The attention mechanism was composed of two identical networks as shown in the center of Fig. 2. The networks were composed of two layers, the first had a size of 2048 units and the second contained several units that matched the output of the layer to which the output attention mask was to be applied. The layer that generated a mask for the CNN output sequence included $90 \times 1296$ units, and the layer that generated a mask for the RNN output sequence included $90 \times 64$ units. The attention-highlighted output of the CNN, $x_{1:T}$, was fed into the RNN which was composed of 90 GRUs, each of 64 units. The output sequence from the RNN was highlighted using the attention mask and fed into the next part that included the fully connected network (the middle right Section of Fig. 2). The attention-highlighted output sequence of the RNN ( $y_{1:T}$) was fed into 4 fully connected layers in order to fuse the temporal features from RNN into the A-P UES opening maximal distension prediction. The first 3 layers were ReLU activated with 128 units and the output layer resembled only one unit with Sigmoid activation that generated the distension prediction value. The two fully connected layers were separated by a dropout layer with a drop rate of 20%.

In this study, we employed the final cost function as the mean squared error between the ground truth values of the A-P UES opening maximal distension ratio to the C2C4 segment length and the predictions generated by the aforementioned network. We used Adam optimizer to train the network due to its superiority in convergence without fine tuning for hyper-parameters [44].

### F. Evaluation

The swallows were randomly divided into 10 equal subsets. A holdout method was used to train the network with swallows 10 times and to test the network with a subset of swallows (also known as 10-fold cross validation). The output from the system was a ratio that represented the normalized A-P UES opening maximal distension with respect to the C2C4 segment length. A previous study with this cohort did not report the ratio to be more than one [21]. The predicted C2C4-normalized UES opening A-P maximal distension was compared to the ground truth using the absolute percentage error (APE) which is defined as follows:

\begin{equation*} APE = \frac {|Prediction - Ground~Truth|\times 100}{Ground~Truth}\end{equation*}

## III. Results

**Fig. 3.**
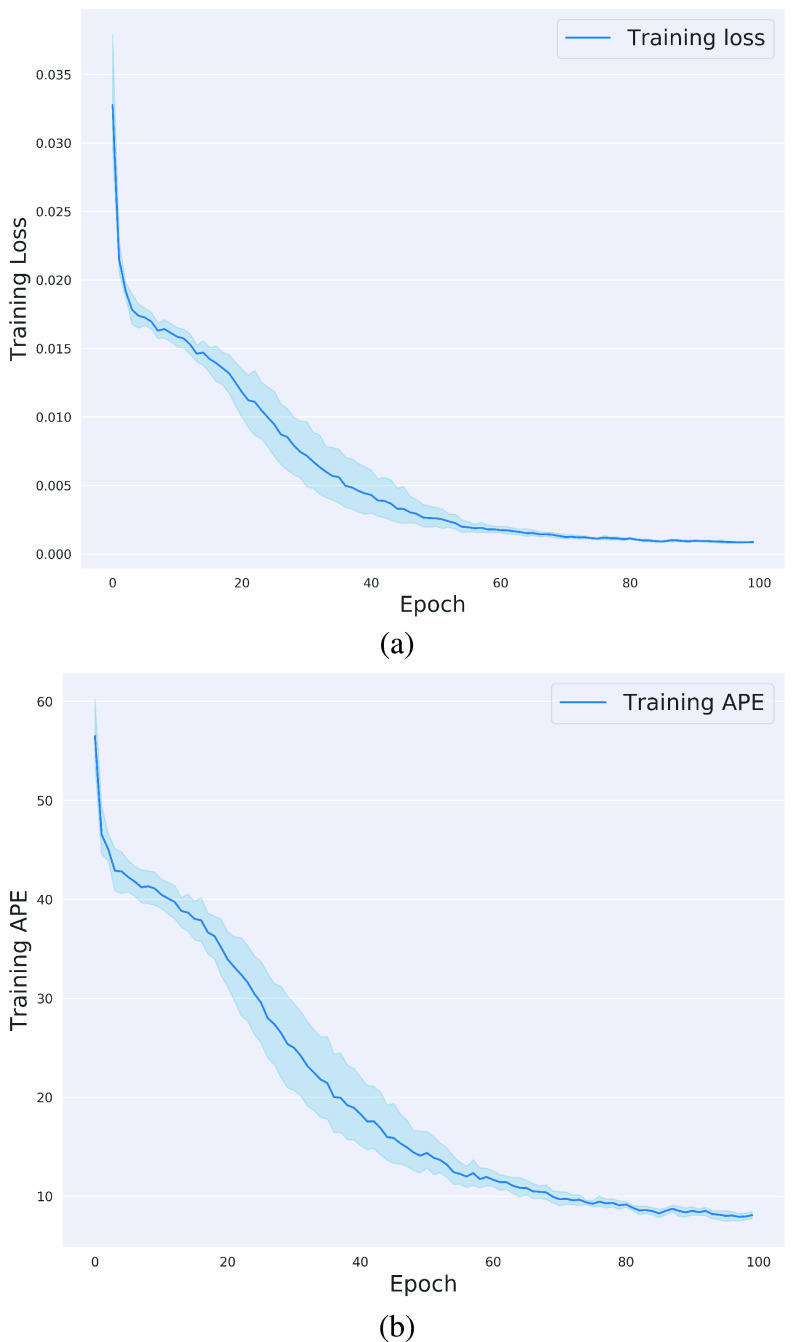
The plots illustrate the progress of the MSE loss function and the APE over the epochs of training the proposed UES opening distension prediction network. (a) represents the MSE loss function over the 100 training epochs across the 10 folds. (b) represents the APE over the 100 training epochs across the 10 folds.

A series of chunks of denoised multi-channel HRCA signals (sizes: $3\times66$) that represented a complete swallow, were fed into the convolutional neural network as shown in Fig. 2. Simultaneously, a zeros/ones mask that represented the UES opening duration, was fed into the fully connected network of the attention generation. The network focused features of the UES opening duration proven to be most associated with UES maximal distension as compared to the features calculated from the entire swallow. Attention was applied in two levels, the first after the last layer of CNN and the second after the last layer of the RNN. The attention-highlighted output was fed into a fully connected network that translated the temporally attention-highlighted features into a normalized A-P UES opening maximal distension prediction. The network was trained over 100 epochs and the evolution of the loss function (MSE) and the absolute percentage error (APE) during training is shown in Fig. 3. The graphs illustrate the MSE and APE during training, which indicate that the network trained well and learned the patterns within the dataset. The results is confirmed by the achieved APE over the validation sets, for which the network produced the normalized UES distension predictions with a mean APE of 27.24± 21.1.

**Fig. 4.**
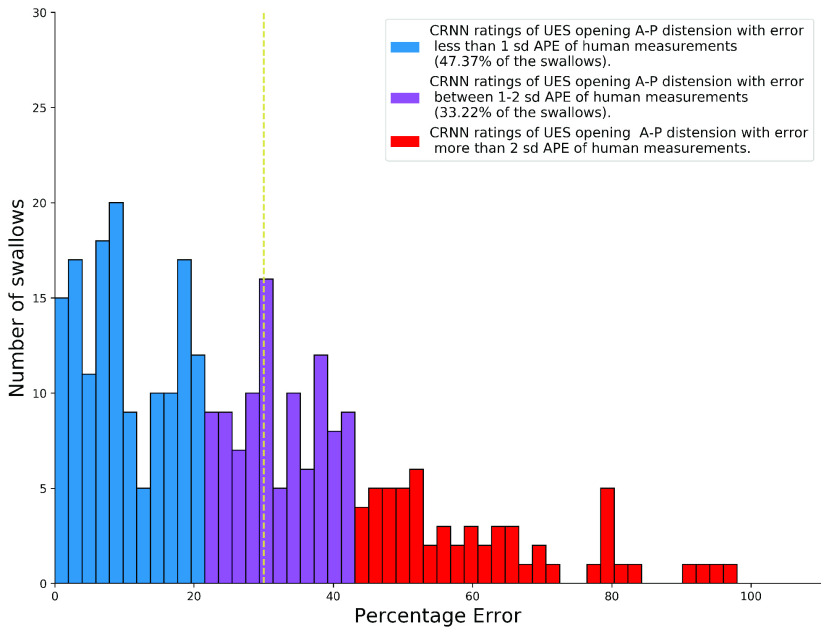
The APE for swallows in the dataset when used in the validation samples. The blue bars represent swallows in which UES opening maximal distension was predicted with an APE of 1 standard deviation, or less, of the entire dataset’s APE as compared to the ground truth labeled by human experts. The purple bars represent swallows in which UES opening maximal distension was predicted with an APE within 1-2 standard deviations of the entire dataset’s APE as compared to the ground truth labeled by expert judges. The red bars represent swallows in which UES opening maximal distension was predicted with an APE of 2 standard deviations or more of the entire dataset’s APE as compared to the ground truth labeled by human experts. The yellow dotted line represents the 30% APE mark; 64.14% of the dataset had swallows with predictions of APE 30% or less.

**Fig. 5.**
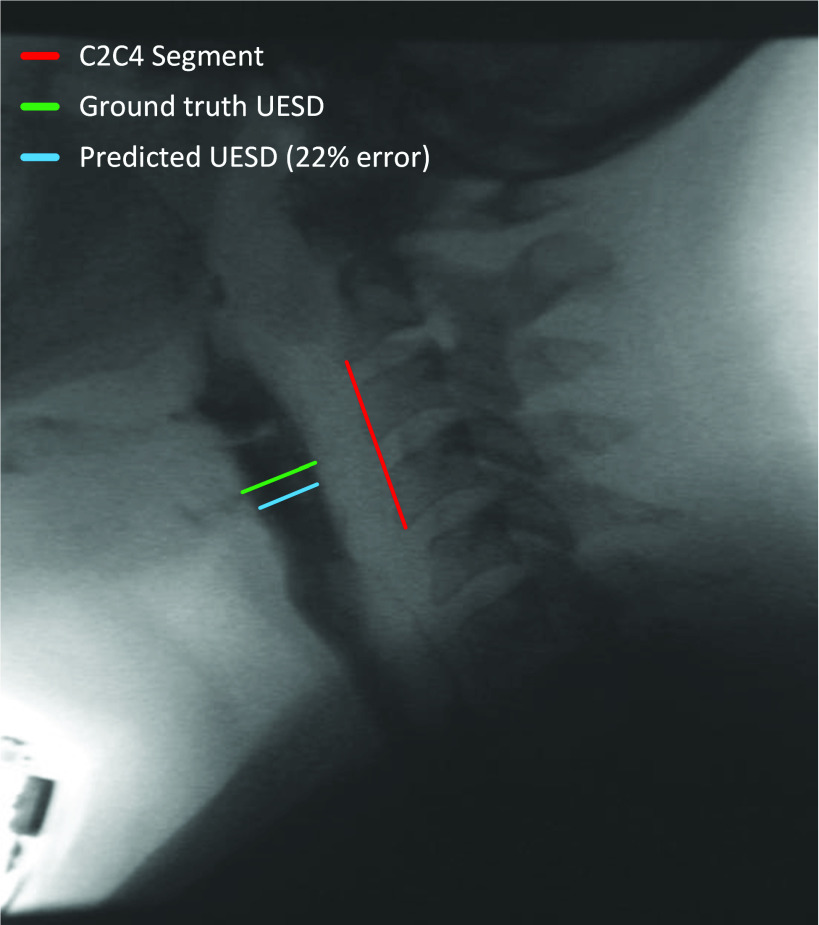
A sample prediction of the C2C4 normalized UES opening maximal A-P distension for a swallow by the proposed system.The green segment represents the ground truth, which measured 0.45 of the C2-C4 length. The light blue segment represents the predicted distension by the network which measured 0.35 of the C2C4 length. The absolute error between the ground truth and the predicted segments is 22% of the ground truth value.

Fig. 4 shows the performance of the proposed UES distension prediction network when using swallows as a testing sample in the validation set. The results show that the prediction network predicted the C2C4 normalized A-P UES opening maximal distension with an absolute error of 30% or less for around 64.14% of the swallows in the dataset, and with an absolute error of 50% or less for around 86.84% of the swallows in the dataset. Fig. 5 shows a sample swallow presented to our proposed system for UES distension prediction. The image depicts a prediction with 22% error (reduction) when compared to the ground truth measured distension. The ground truth for this swallow measured approximately 0.45 of the C2C4 segment length and the predicted segment measured approximately 0.35 of the C2-C4 segment length.

## IV. Discussion

The primary goal of this study was to determine the feasibility of using HRCA vibratory signals as input for a deep learning architecture to non-invasively predict UES opening maximal anterior- posterior distension. We presented a hybrid deep neural network model that used CNNs, RNNs, and attention mechanisms to extract local features from raw HRCA vibratory signals. The model temporally correlated and adjusted the features to accurately predict the value of the A-P UES maximal distension. The results showed that HRCA combined with deep learning can fairly accurately predict the C2-C4 normalized A-P UES opening maximal distension when compared to the ground truth distension labeled by expert human judges.

The deep learning architecture employed in this study was motivated by previous studies that investigated the correlation between HRCA signals and UES opening duration and A-P UES maximal distension [13], [3], [21]. These studies presented multiple findings that inspired the design for the architecture used in this study. The first significant finding was that HRCA signals are highly correlated with UES opening duration and can be used with deep learning to predict the exact time of UES opening and closing [3], [13]. The second finding was that the correlation between the HRCA signal features and A-P UES maximal distension is the strongest during UES opening. This finding guided us to use attention mechanisms to focus on key features during the swallow [21].

Our proposed network predicted the C2-C4 normalized UES distension with an error percentage of 30% or less for more than half of the swallows (64.14%) and less than 50% for 86.84% of the swallows in the dataset. The error rates achieved in this study are comparable to common error rates between humans for similar measurements such as hyoid bone labeling to track hyoid bone displacement [19]. In the study of tracking hyoid bone displacement, raters placed anchors on the anterior-inferior and posterior-superior corners of the hyoid bone. These points were used to construct a bounding box around body of the hyoid. The overlap between the bounding boxes marked by different raters for the same swallows never exceeded 79.09% of the hyoid bone body [19].

The results of our proposed prediction system are noteworthy because the system performed well despite lack of exact agreement between human raters. Human judgments are inherently subjective and the quality and resolution of x-ray images from VFSS, and differences in machines used for judgments increase variability. It is difficult for humans to distinguish precise pixels, and even a few pixels difference could lead to a large change in the orientation and length of a measured segment. Given the variability and errors in human measurements, the performance of our network can be considered acceptable; however, we also expect that the performance and generalizability could be enhanced by using a larger dataset of swallows which is one of the future directions of the study.

The future directions of this study also include enhancing the prediction performance of the network using multi-task learning to train a prediction framework to simultaneously predict UES opening and closure (i.e., opening duration) and the maximal A-P distension. Such a model would use shared representations to quickly learn the common features between the downstream prediction tasks, could reduce overfitting, and would increase data efficiency because of shared information between the prediction tasks.

Clinically, non-invasive estimation of UES distension could support efficient diagnosis and rehabilitation of swallowing disorders. For example, this type of system could be used as a biofeedback tool. Patients could use the system during treatment to determine whether they are performing rehabilitative swallow “maneuvers” correctly. The more effectively they can prolong UES duration or enhance distention, the less likely they are to have post-swallow residue, which can lead to aspiration. Including non-invasive estimations of UES distention in swallowing assessments could reduce the cost of dysphagia management by limiting the need for advanced diagnostic imaging studies such as VFSS. Non-invasive estimation of UES distension could also reveal acceptable ranges of normal/healthy UES distention, thus helping to identify patterns that deviate from the norm. Furthermore, it can be used to track the deterioration of this aspect of swallowing function in relevant patient populations such as patients with neurodegenerative diseases.

## V. Conclusion

In conclusion, this study proposed a new method to use HRCA signals to non-invasively estimate the anterior-posterior UES opening maximal distension during swallowing. First, we simultaneously collected VFSS images and HRCA signals. Then, we developed a protocol for human raters to judge the UES maximal A-P distension in VFSS images. The resulting measurements were used as the ground truth. We employed a hybrid deep neural network that used CNNs, RNNs, and attention mechanisms to perform predictions of UES opening maximal distention from the raw HRCA signals. The results revealed that HRCA combined with deep learning models can provide a fairly accurate estimate of the A-P UES maximal distension during swallowing when compared to the ground truth. This study, along with other studies investigating the correlations between HRCA signals and swallowing kinematics, provides evidence that HRCA combined with advanced signals processing techniques has the power to provide non-invasive, time-efficient, and low cost diagnostic value for dysphagia assessment and management.
